# MicroRNA expression profile in retina and choroid in oxygen-induced retinopathy model

**DOI:** 10.1371/journal.pone.0218282

**Published:** 2019-06-12

**Authors:** Michel Desjarlais, Jose Carlos Rivera, Isabelle Lahaie, Gaël Cagnone, Maëlle Wirt, Samy Omri, Sylvain Chemtob

**Affiliations:** 1 Department of Ophthalmology, Maisonneuve-Rosemont Hospital Research Center, University of Montréal, Montréal, Québec, Canada; 2 Departments of Pediatrics, Ophthalmology and Pharmacology, Centre Hospitalier Universitaire Sainte-Justine Research Center, Montréal, Québec, Canada; Children's Hospital Boston, UNITED STATES

## Abstract

**Background:**

Ischemic retinopathies (IRs) are leading causes of visual impairment. They are characterized by an initial phase of microvascular degeneration and a second phase of aberrant pre-retinal neovascularization (NV). microRNAs (miRNAs) regulate gene expression, and a number play a role in normal and pathological NV. But, post-transcriptional modulation of miRNAs in the eye during the development of IRs has not been systematically evaluated.

**Aims & methods:**

Using Next Generation Sequencing (NGS) we profiled miRNA expression in the retina and choroid during vasodegenerative and NV phases of oxygen-induced retinopathy (OIR).

**Results:**

Approximately 20% of total miRNAs exhibited altered expression (up- or down-regulation); 6% of miRNA were found highly expressed in retina and choroid of rats subjected to OIR. During OIR-induced vessel degeneration phase, miR-199a-3p, -199a-5p, -1b, -126a-3p displayed a robust decreased expression (> 85%) in the retina. While in the choroid, miR-152-3p, -142-3p, -148a-3p, -532-3p were upregulated (>200%) and miR-96-5p, -124-3p, -9a-3p, -190b-5p, -181a-1-3p, -9a-5p, -183-5p were downregulated (>70%) compared to controls. During peak pathological NV, miR-30a-5p, -30e-5p and 190b-5p were markedly reduced (>70%), and miR-30e-3p, miR-335, -30b-5p strongly augmented (by up to 300%) in the retina. Whereas in choroid, miR-let-7f-5p, miR-126a-5p and miR-101a-3p were downregulated by (>81%), and miR-125a-5p, let-7e-5p and let-7g-5p were upregulated by (>570%) during NV. Changes in miRNA observed using NGS were validated using qRT-PCR for the 24 most modulated miRNAs. *In silico* approach to predict miRNA target genes (using algorithms of miRSystem database) identified potential new target genes with pro-inflammatory, apoptotic and angiogenic properties.

**Conclusion:**

The present study is the first comprehensive description of retinal/choroidal miRNAs profiling in OIR (using NGS technology). Our results provide a valuable framework for the characterization and possible therapeutic potential of specific miRNAs involved in ocular IR-triggered inflammation, angiogenesis and degeneration.

## Introduction

Ischemic retinopathies (IRs) such as retinopathy of prematurity (ROP) and diabetic retinopathy (DR) remain the leading causes of visual impairment and blindness worldwide^1^. IRs are characterized by an initial phase of microvascular degeneration followed by ischemia and a subsequent phase of pathological pre-retinal neovascularization (NV). Several mechanisms that control microvascular development and vascular repair are altered during IRs [1,2] including those related to the expression of microRNAs (miRNAs), a family of non-coding RNAs (20–25 nucleotides) involved in post-transcriptional regulation of genes [3,4]. miRNAs regulate a wide range of physiological and pathological process [5,6]. miRNAs can repress their targets by inhibiting protein translation or by degrading specific mRNA with a perfectly complementary target binding sequence (miRNA/mRNA) [6]. It is estimated that miRNAs negatively regulate the expression of more than 60% of genes involved in various cellular processes such as oxidative stress, apoptosis/survival, autophagy, inflammation, cell migration, proliferation and growth [7]. As anticipated, the level of miRNA expression differs between cells types and pathological states [5–7], suggesting a critical role of miRNAs in the progression of various diseases including cancer [7], cardiovascular diseases [8], degenerative diseases [9], and retinopathies [3,10].

miRNAs are key regulators of blood vessel development [11] and have been implicated in pathological retinal and choroidal neovascularization [10]. But the role of endogenous miRNAs in IRs (mostly as it applies to the choroid) remains poorly understood. This is particularly relevant since modulation of miRNA activity with mimics and antagomiRs has potential therapeutic value [4,11–13]. Therefore, using the advanced next generation sequencing (NGS) technology [14,15] we evaluated the miRNA expression profile in the retina and choroid in the IR model of oxygen-induced retinopathy (OIR), and proceeded to predict targets associated with abnormal vascular development.

## Materials and methods

### Animal care

All animal experimental procedures were performed with strict adherence to the ARVO Statement for the Use of Animals in Ophthalmic and Vision Research and approved by the Animal Care Committee of the Hospital Maisonneuve-Rosemont in accordance with guidelines established by the Canadian Council on Animal Care.

### Oxygen-induced retinopathy model in rats

**Fig 1:**
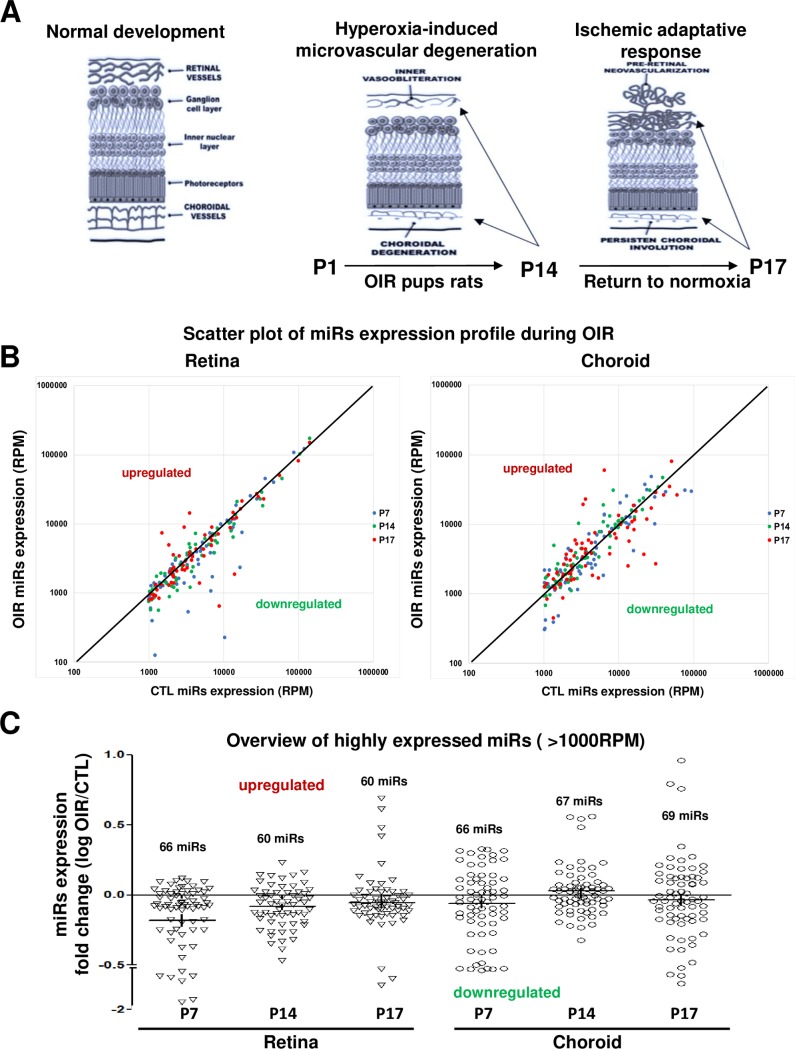
Overview of differential-expression profile of miRNAs in the retina and choroid of rats subjected to OIR. **A**. Schematic representation of OIR-induced pathological effects on microvascular development in the retina and choroid at different postnatal ages (P). Hyperoxia exposure leads to vessel degenerations (P1 to P14). At P17 (return to normal room air for 3 days), one observes pathological neovascularization (NV) pre-retinally associated with persistent vessel degeneration in the choroid. **B and C**. Scatter plot of miRNA expression alteration during OIR (RPM) (**B**) and overview of highly expressed miRNAs (more than 1000 reads per million [RPM]) in retinal and choroidal tissues and their differential-expression profile (log fold change: OIR/CTL) (**C**) assessed by next generation sequencing (NGS). Data are log RPM fold change ratio (OIR/CTL) of 5 rats/group.

We used an established model of oxygen-induced retinopathy (OIR) in rats [16], to evaluate the miRNAs expression profiles in retinal and choroidal tissues during the pathological progress of this disease. This model is characterized by a first phase of progressive microvascular degeneration that occurs during cycling oxygen (50% - 10% every 24 hours) between days 1 and 14, followed by a second phase of abnormal pathological NV that take place when pup rats are return to room air between days 14 and 17 (**S1 Fig and Fig 1A**). Approximately 4 h after birth, litters of Sprague-Dawley albino rats (Charles River, St. Constant, QC, Canada) were placed with their mothers in an oxygen-regulated environment (OxyCycler A820CV; BioSpherix, Ltd., Redfield, NY,USA) adjusted to alternate between 50% and 10% oxygen every 24 hours for 14 days (OIR group). At postnatal (P) day 14, rat pups were transferred to room air (21% O_2_) for 3 days (P17) [17]. Age-matched normoxic control rat pups (CTRL) were kept in room air (21% O_2_) throughout the experiment. OIR and CTRL rat pups were anesthetized with isoflurane (2%) and euthanized by decapitation at P7, P14 and P17; these ages correspond respectively to early vascular degeneration, end of vascular degeneration phase and pre-retinal NV in the OIR model. Eyes were immediately enucleated for retinal and choroidal dissection, and rapidly processed for RNA extraction using TRIZOL according to the manufacturer’s protocol (Invitrogen, Thermo Fisher Scientific Corporation, Carlsbad, CA, USA).

### miRNA isolation and next generation sequencing analyses

Total RNA was extracted from retinas and choroid tissues from OIR and CTRL groups at P7, P14 and P17, using the miRNeasy mini kit (Qiagen) according to the manufacturer’s protocol. Quantification of total RNA was made with a nanodrop and 1 μg of total RNA was used for library preparation. Quality of total RNA was assessed with the BioAnalyzer Nano (Agilent) and all samples had a RIN above 8. Library preparation was done with the Truseq Small RNA library preparation kit (Illumina, Cat no. RS-200-0012). Eleven PCR cycles were required to amplify libraries. Libraries were quantified with a nanodrop and the quality was assessed with the BioAnalyzer High Sensitivity (Agilent). All libraries were diluted to 10 nM, normalized and pooled (n = 5) to equimolar concentration based on Miseq v2 50 cycles using 7pM of pooled library. Sequencing was performed with the Illumina Hiseq2000 using the Hiseq Reageant Kit v3 (200 cycles, pairedend) and 1.7 nM of the pooled library. Around 70 million paired-end reads were generated per sample. Quantification includes the raw read count, as well as normalized expression level as RPM values (reads per million reads mapped) to account for the variability in the library size [12].

### miRNA differential-expression profiling data analyses

To identify the differentially miRNAs expression in retinas and choroids tissues at different stage of OIR, NGS analyses generates 12 smalls RNA libraries to compare the total of all expressed miRNA in CTL rats’ pup’s vs OIR rats’ pups at P7 (initial vascular degeneration phase), P14 (peak of retinal vasoobliteration and choroidal involution) and P17 (retinal NV and persistent choroidal involution). Approximatively 15 million RNA sequences were read per sample and around 10 million of them correspond to miRNAs of generated libraries (**S1 Table**). The total of specific individual miRNAs reads was normalized by the total of sequences reads per million of reads mapped (RPM). Based on the high-throughput of miRNA sequencing data generate, we next performed differential-expression profiling analyses by first removed (cut off) the miRNAs with low expression level (RPM <1000) [12]. Next, we selected arbitrary modulation threshold ratio (OIR/CTL) in function of the number of miRNAs modulated in each time points in choroid and retinal tissues. miRNAs were considered to be significantly up- or down regulated if the modulation ratio (OIR/CTL) were greater than 1.2-fold (20% of modulation) or highly express in tissues if the RPM where greater than 1000.

### qRT-PCT validation analysis of miRNAs profiling

Total RNA extracted from retinas and choroid tissues from OIR and CTRL groups at P7, P14 and P17, using the miRNeasy mini kit (Qiagen) according to the manufacturer’s protocol was reverse transcribed using with miScript II RT kit (Catalogue # 218161, QIAGEN, Hilden, Germany) according to manufacturer’s guidelines. Real-time PCR for mature miRNA expression validation was performed using 25 ng of cDNA sample by quantitative real-time PCR using iTaq Universal SYBR Green Supermix (BioRad) with 2 μM of specific primers for the selected miRNAs designed using Primer Bank and NCBI Primer Blast software (Alpha DNA, Montreal, Canada). The amplification level was programmed with an initial step of 20 sec at 95°C, followed by 40 cycles of 1 sec at 95°C and 20 sec at 60°C. Relative expression (RQ = delta/delta CT) was calculated using quantitative analysis of miRNAs expression generated using a sequence detection system (ABI Prism 7500; Applied Biosystems, Foster City, CA, USA) and normalized to U6 snRNA.

### Identification of potential predictive targets of OIR-modulated miRNAs

Potential predicted and validated angiogenic, inflammatory and apoptotic target genes of selective OIR-modulated miRNAs were analyzed according to the bioinformatical algorithms of miRSystem database (http://mirsystem.cgm.ntu.edu.tw/index.php) which integrates seven well known miRNAs target genes prediction programs [18]: DIANA, miRnanda, miRBridge, PicTar, PITA, rna22 and TargetScan. Total hit represents the number of target gene prediction program that identify the selected target gene (mRNA) as a predictive target for the miRNA.

### Immunohistochemistry of retinal and choroidal vessels

For retinal vasculature, retinal flat mount dissection was performed on the enucleated eyes fixed in 4% paraformaldehyde for 1 hour at room temperature and then stored in PBS until used. The retinas were incubated overnight in 1% Triton X100, 1 mM CaCl2/PBS with the tetramethylrhodamine isothiocyanate–conjugated lectin endothelial cell marker Bandeiraea simplicifolia (1:100; Sigma-Aldrich Corp., St. Louis, MO, USA). Retinas were washed in PBS and mounted on microscope slides (Bio Nuclear Diagnostics, Inc., Toronto, ON, Canada) under coverslips with mounting media (Fluoro-Gel; Electron Microscopy Sciences, Hatfield, PA, USA). Retinas were photographed under an epifluorescence microscope (Zeiss AxioObserver; Carl Zeiss Canada, Toronto, ON, Canada), and the images were merged into a single file using the MosiaX option in the AxioVision 4.6.5 software (Zeiss). For choroidal vasculature, retinal cross-sections were performed. Eyes were collected, dehydrated by alcohol, and embedded in paraffin. Sagittal sections (7 μm thick) were cut by microtome (RM 2145; Leica, Wetzlar, Germany). Posterior eyecups were frozen in optimal cutting temperature medium and stained for choroidal vessels with TRITC-conjugated tetramethylrhodamine isothiocyanate-labeled lectin (Sigma-Aldrich) in the cryosections. Sections were then visualized with an epifluorescence microscope (Eclipse E800; Nikon, Tokyo, Japan).

### Statistical analysis

miRNAs validation results are mean ± SEM. Statistical significance was evaluated by two-way ANOVA followed by a Bonferroni post hoc test. A value of *P<*0.05 was interpreted to denote statistical significance.

## Results

### miRNA expression profile in the rat OIR model

To identify the differences in miRNA expression during OIR, we employed next generation sequencing (NGS) technology to analyze miRNAs profile in total RNA isolated from retinal and choroidal tissues from rat pups (CTR and OIR) at P7, P14 and P17. NGS analysis revealed an average of 10 million miRNAs raw read sequences of between 15–20 million total raw reads per sample (**S1 Table**). When miRNAs were normalized on RPM value (reads per million of reads mapped) corresponding to the number of specific miRNA raw read (UMI)/number of total raw read, we detected ~1000 miRNAs in both tissues (retina and choroid) with a highly variable level of expression (data not show). We focused on the most abundant retinal/choroidal-miRNAs by establishing arbitrary a cut-off with minimum 1000 RPM in miRNA expression levels as shown in **Fig 1B** (Scatter plot of differencial-expression). Our results showed a range of 60 to 69 miRNAs highly expressed (>1000 RPM) in the retina and choroid at the different postnatal time points (**Fig 1C**), representing approximately 6% of the total miRNAs expressed. Interestingly, we observed a global miRNA downregulation pattern in retinal samples from OIR-subjected animals, with a peak at P7, and a trend to normalization at P17. While in the choroid, OIR was associated with a small downregulation of miRNAs at P7 and P17 and a general upregulation at P14.

### miRNA expression in the early phase of OIR

**Fig 2:**
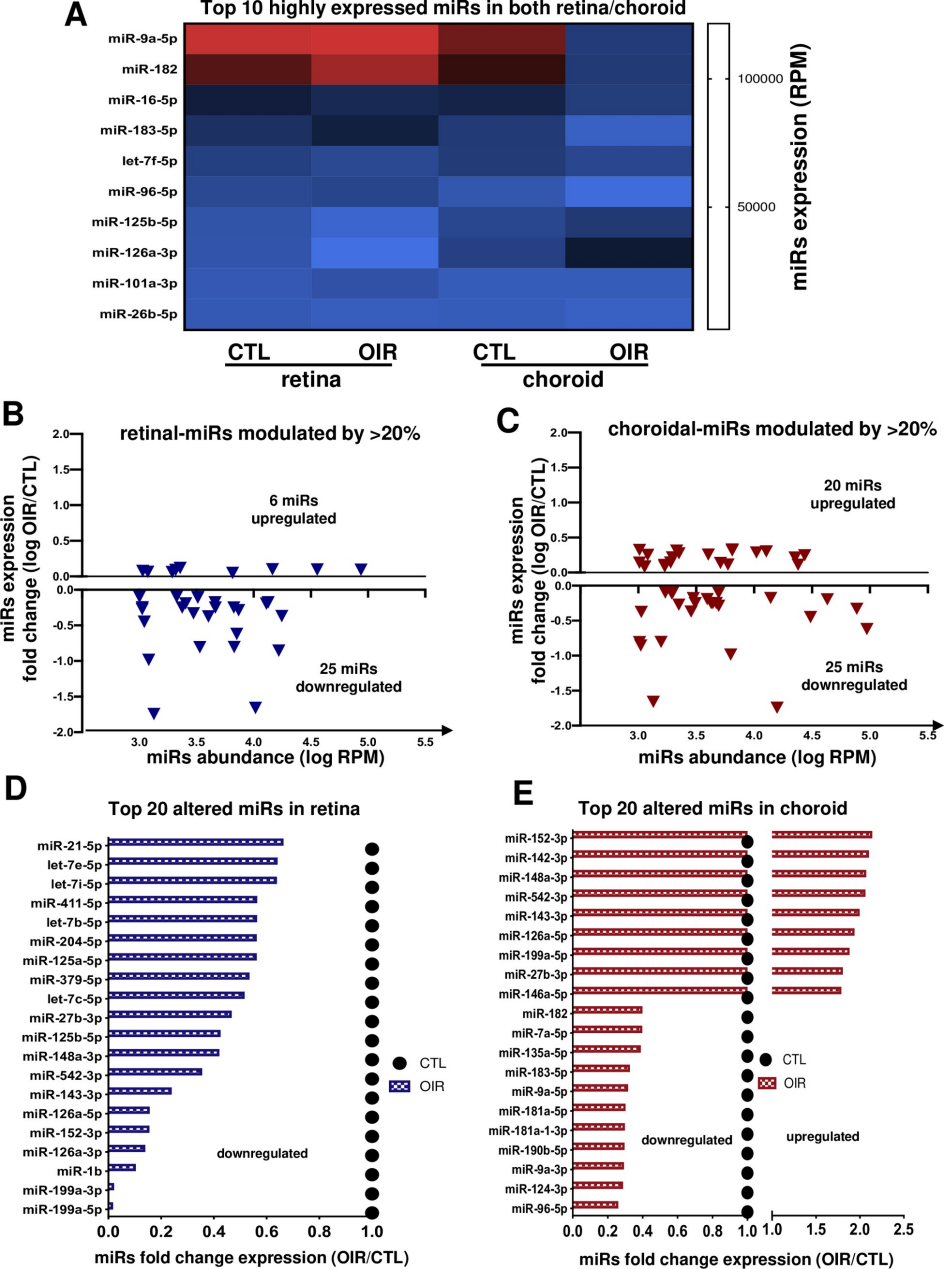
miRNA expression profile during OIR-induced vessel degeneration (at P7). **A**. Heat map of 10 most expressed miRNAs in retinal and choroidal tissues. **B and C.** Overview of most abundant miRNA (more than 1000 RPM) modulated by OIR (1.2 fold or more) in the retina (**B**) and choroid (**C**). **D and E.** Summary of top 20 OIR-modulated miRNAs in the retina (**D**) and choroid (**E**). Data are fold change ratio (OIR/CTL) or log fold change, total RPM or log RPM of 5 rats/group.

In the early vasoobliterative phase of OIR (at P7) NGS analysis showed miR-9a-5p, -182, -16-5p, -183-5p, let-7f-5p as the most abundant miRNAs present in both retinal and choroidal tissues during this phase (**Fig 2A**); miR-9a-5p and miR-182 exhibited a remarkable level of expression above 100000 RPM in the retina and 75000 RPM in the choroid. To examine miRNAs profiling signature in early stage of vessel degeneration during OIR, we focused on miRNAs modulated by 1.2-fold (20% of modulation) relative to corresponding tissues of normoxia-raised rats (controls); we established this arbitrary cut-off based on both the minimal/maximal miRNA expression level as previously described [12]. Analysis identified 31 miRNAs altered in the retina of which 25 were downregulated and 6 were upregulated (**Fig 2B**). In the choroid, 45 miRNAs were 20% modulated up (20) or down (25) relative to controls (**Fig 2C**). Of the 20 miRNAs most modulated in retina all were downregulated, as most notably 6 of them (miR-199a-3p, -199a-5p, -1b, -126a-3p) displayed a >80% suppression (**Fig 2D**). On the other hand in the choroid, 9 of the 20 miRNAs most altered were upregulated including 4 miRNAs (miR-152-3p, -142-3p, -148a-3p, -532-3p) substantially upregulated >200% (**Fig 2E**), and 11 were downregulated, among them 8 miRNAs (miR-96-5p, -124-3p, -9a-3p, -190b-5p, -181a-1-3p, -9a-5p, -183-5p) by >70% (**Fig 2E**).

### miRNA expression profile at the end of vasoobliterative phase of OIR

**Fig 3:**
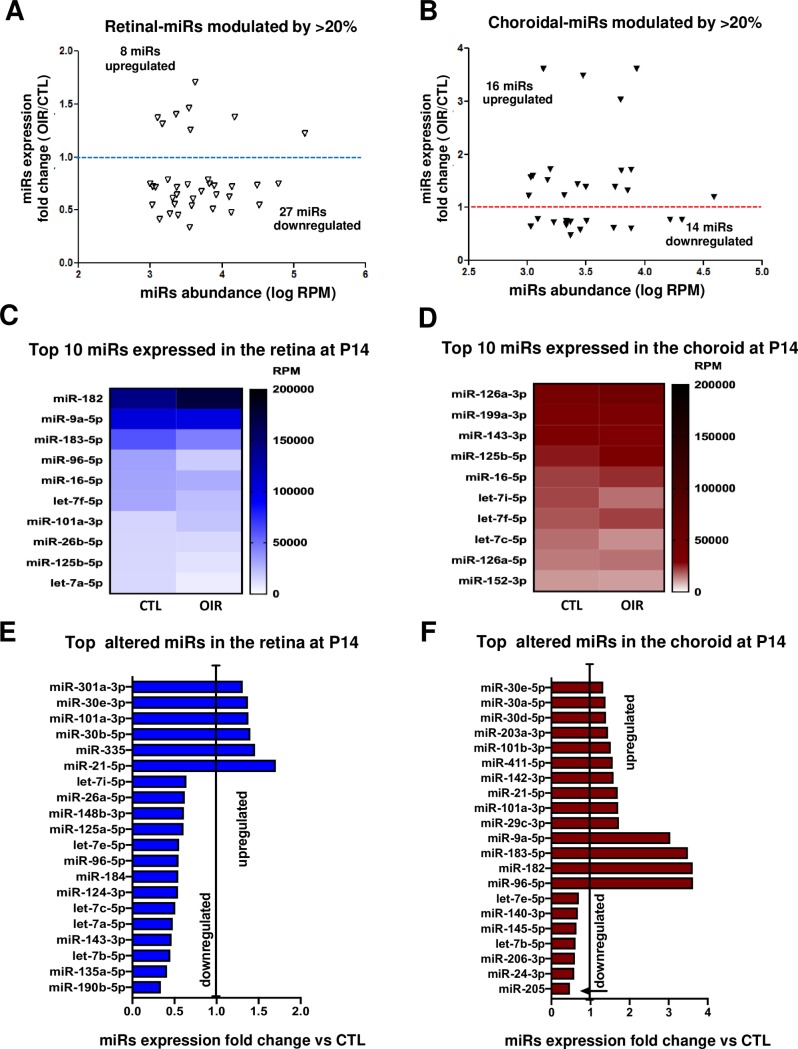
miRNA expression profile at end of retinal vasoobliteration phase in OIR (P14). **A and B.** Overview of most abundant miRNA (more than 1000 RPM) modulated by OIR (1.2 fold or more) in the retina (**A**) and choroid (**B**). **C- F.** Summary of top most expressed miRNAs in the retina (**C**) and choroid (**D**), and most modulated miRNAs by OIR in the retina (**E**) and choroid (**F**). Data are fold change ratio (OIR/CTL), total RPM or log RPM of 5 rat pool/group.

Next, we analyzed the miRNA expression profile at the end of the vasoobliterative phase at P14 [19]. 35 miRNAs in the retina (8 upregulated and 27 downregulated) and 30 miRNAs in the choroid (16 upregulated and 14 downregulated) were modulated by >20% in OIR relative to controls and were expressed more than 1000 RPM (**Fig 3A and 3B**). Among the highly expressed miRNAs (l000 RPM > 4.0), only 6 miRNAs (retina: miR-190b, -135a, let-7b-5p, -143-3p, let-7a-5p; choroid: miR-205) were downregulated by ~50% (**Fig 3E and 3F**). Many more miRNAs were upregulated at P14, including miR-21-5p that was increased by more than 1.7-fold in the retina and miR-9a-5p, -183-5p, -182, -96-5p increased more than 3.0-fold in the choroid (**Fig 3E and 3F**). miR-182, -95-5p and -9a-5p were the most abundant miRNAs in retina at P14 (**Fig 3C**) and miR-126a-3p and -199a-3p in the choroid but these were not modulated (**Fig 3D**); latter miRNA was also most abundant in both retina and choroid at P7 (**Fig 2D**). So far, our data suggest that the most significant miRNAs alterations were observed in the early events of OIR at P7.

### miRNA profile during retinal NV phase (and persistent choroidal involution)

**Fig 4:**
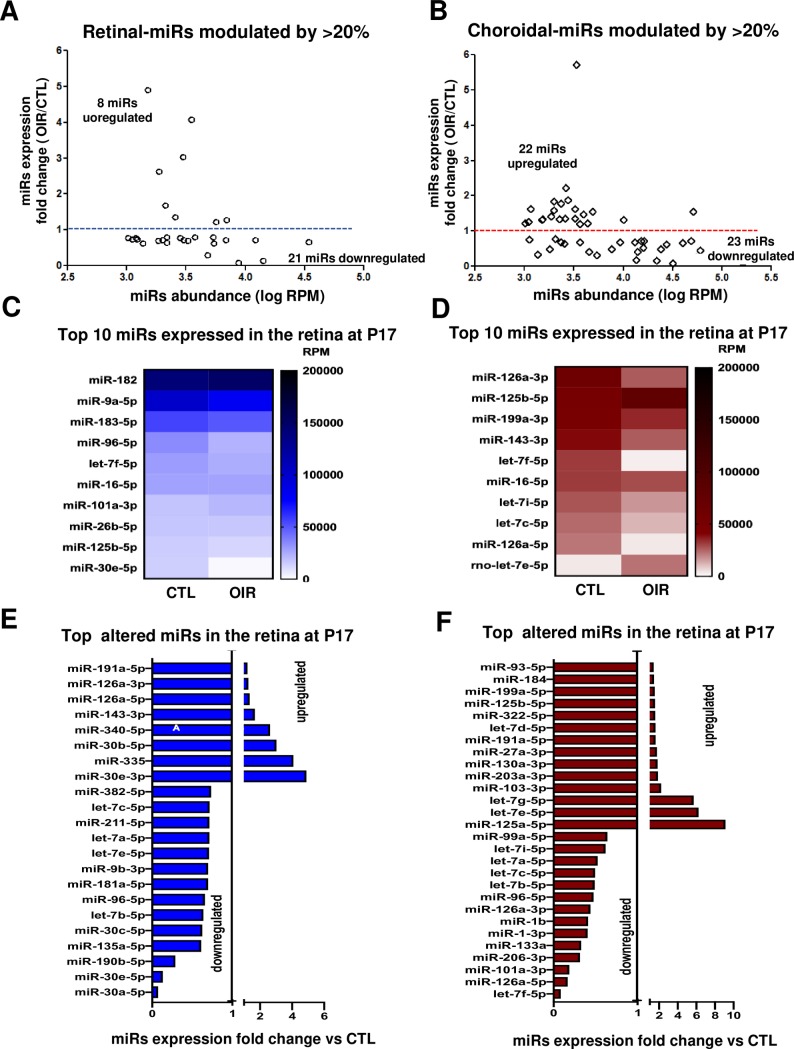
miRNA expression profile during pre-retinal NV (and persistent choroidal involution). A and B. Overview of most abundant miRNA (more than 1000 RPM) modulated by OIR (1.2 fold or more) in the retina (A) and choroid (B) during the NV phase (P17). C- F. Summary of top most expressed miRNAs in the retina (C) and choroid (D), and most modulated miRNAs by OIR in the retina (E) and choroid (F). Data are fold change ratio (OIR/CTL), total RPM or log RPM of 5 rats/group.

During the NV phase NGS analysis revealed 45 miRNAs modulated by ~20% (>1000 RM) in the choroid and 29 miRNAs in the retina (**Fig 4A and 4B**). 72% of these miRNAs in the retina were downregulated, whereas in the choroid the approximate number of up- and downregulated miRNAs was comparable. In the retina, 3 miRNAs were markedly downregulated: miR-30a-5p (fold change [fc] = 0.073), miR-30e-5p (fc = 0.132) and miR-190b-5p (fc = 0.289), and 3 miRNAs strongly upregulated by ~3-fold: miR-30e-3p (fc = 4.896), miR-335 (fc = 4.083), miR-30b-5p (fc = 3.024) (**Fig 4E**). Interestingly, almost all these miRNAs altered in the retina were isoforms that belong to the miR-30 family, whose members play an important role in tissue and organ development and have been associated with cancer [20]. In the choroid 6 miRNAs were strongly modulated including let-7f-5p (fc = 0.085), miR-126a-5p (fc = 0.167) and miR-101a-3p (fc = 0.185), as these were found to be downregulated; miR-125a-5p (fc = 9.13), let-7e-5p (fc = 6.233) and let-7g-5p (fc = 5.714) were upregulated (**Fig 4F**). Interestingly, half these miRNAs in the choroid belong to the let-7 family, implicated in several biological and pathological diseases process such as embryogenesis, tumor suppressor and angiogenesis [21]. Three of the most abundant miRNAs in the retina during NV phase were miR-96-5p (fc = 0.657), miR-30e-5p (fc = 0.132) and let-7a-5p (fc = 0.716) (**Fig 4C**), and in the choroid were miR-126a-3p (fc = 0.439), miR-125b-5p (fc = 1.555) and let-7f-5p (fc = 0.85) (**Fig 4D**). Based on their known functions, our findings suggest that miR-30 and the let-7 families could participate in chorioretinopathies.

### Validation of most modulated miRNAs identified by NGS in the retina and choroid during the different phases of OIR

**Fig 5:**
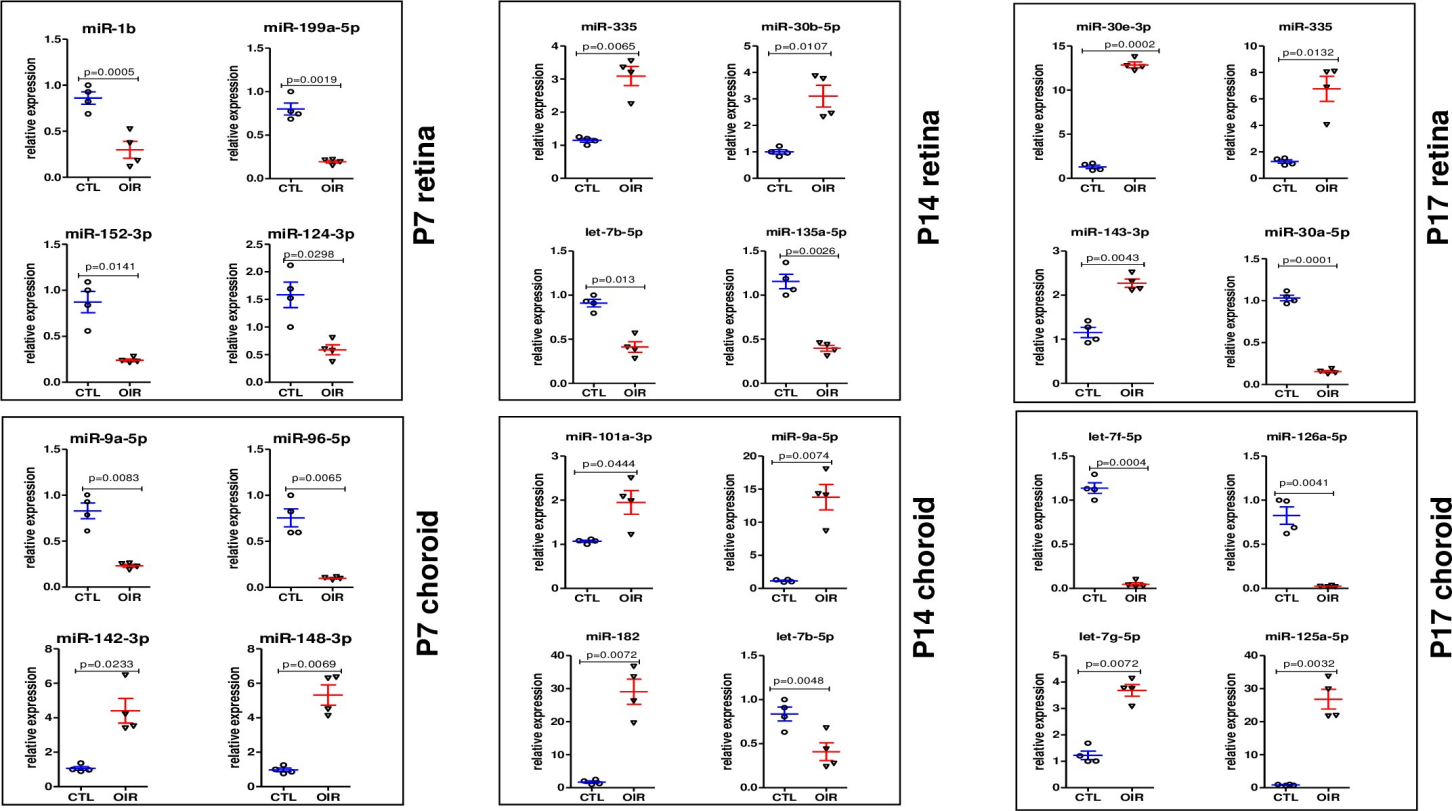
qRT-PCR validation analysis of the top most OIR-modulated miRNAs previously indentified by NGS. qRT-PCR validation of 24 miRNAs most modulated by OIR. Data show individual and mean values of miRNA expression normalized on U6 for each animal; P value was calculated by two-way anova with Bonferroni post-hoc test (n = 4).

To verify and validate NGS results, we performed qRT-PCR analyses of the 24 miRNAs most up- or down-regulated in the retina and choroid at the different phases of OIR, including 8 miRNAs by time points at P7, P14 and P17. As shown in **Fig 5**, we found a similar downregulation or upregulation expression level mean ratio (OIR/CTL) with individual animal value as observed previously by NGS. In the retina, 7 miRNAs were significantly (P<0.05) downregulated and 5 significantly upregulated. Among these retinal miRNAs, miR-1b and miR-30a-5p were strongly downregulated respectively at P7 and P17 (P<0.0001). In addition, 5 of the selected upregulated miRNAs were significantly increased in OIR retina with (P<0.01). Among these, miR-30e-3p and miR-335 displayed the greatest upregulation (>7-fold compared to CTL). In the choroid 5 significantly downregulated miRNAs were confirmed, including miR-95-5p (P<0.0065) at P7, and miR-126a-5p (P = 0.0041) and let-7f-5p (P = 0.0004) at P17 were robustly supressed. Among the 7 upregulated miRNAs validated in the choroid, we found that miR-182 (P = 0.0072) at P14 and miR-125a-5p (P = 0.0032) at P17 displayed a >20-fold increase compared to CTL.

### Identification of miRNAs associated with angiogenesis, inflammation and apoptosis during OIR

**Fig 6:**
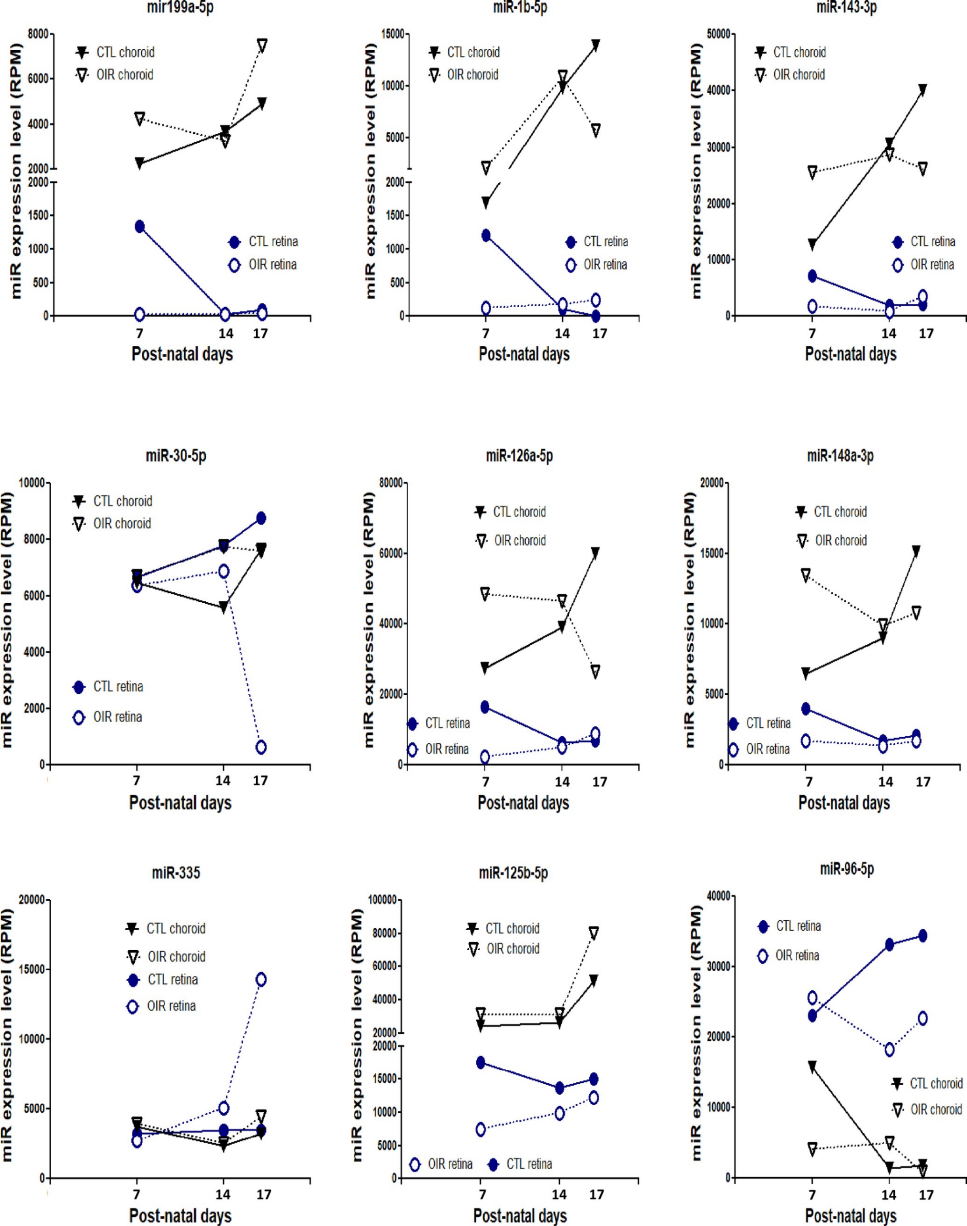
Age-dependent expression pattern of 9 miRNA associated with angiogenesis and most modulated by OIR. Retinal and choroidal tissues were collected at P7, 14 and 17 in OIR and control (normoxia-raised) rats. Data are total RPM of a 5 rats/group.

We next evaluated the expression pattern of 9 miRNAs most modulated during OIR (**Fig 6**) and previously shown to exert a significant impact on angiogenesis (**S2 and S3 Tables**). Expression levels of miRNA as a function of time differed markedly, and some miRNAs exhibited opposing profiles between retina and choroid, as seen for miR-335 and -96-5p **(Fig 6**).

We proceeded to compile relevant information from the literature associated with anti- and pro-angiogenic activities of these 9 miRNAs highly altered in OIR. 4 miRNAs revealed to exhibit pro-angiogenic functions (miR-1b-5p, -30a-5p, -126a-5p, 96-5p), 2 miRNAs anti-angiogenic functions (miR-199a-5p, -125b-5p) and 3 miRNAs showed an unclear or contradictory function/effect on angiogenesis (miR-143-3p, -148a-3p, -335) (**S2 and S3 Tables**). Among the pro-angiogenic miRNAs, miR-1b-5p induces cardiomyocyte progenitor cells by targeting Spred1 [22]; miR-30a-5p regulates endothelial tips cell formation by modulating Notch signaling [23]; miR-126a-5p an endothelial specific miRNA is known to be required for vascular integrity, embryogenesis and angiogenic activation signaling [24,25]; miR-96-5p, a miRNA induced in hypoxic condition [26], plays a pro-angiogenic role in carcinogenesis [27]; miR-199a-5p targets ETS-1 to suppress HMEC angiogenesis [28] and suppresses tumor growth by inhibiting VEGF and HGF signaling [29]; miR-125b-5p has been inversely correlated with VEGF expression [30].

In addition to angiogenesis, we evaluated links of these 9 OIR-modulated miRNAs to inflammation and apoptosis. Results indicate that these miRNAs have the properties to modulate inflammatory and apoptotic signaling in different cell types (**S4 Table**). Seven of theses miRNAs exert anti-inflammatory effects, such that miR-199a-5p suppresses IKKb/NF-κB/IL-8 in endometrial stromal cell [31] and COX-2 in osteoarthritic chondrocytes [32]; miR-1 inhibits lung TH2 inflammation-induced by ovalbumine [33]; miR-143 inhibits IL-13 in epithelial cell [34]; miR-30a antagonizes the action of INFγ in adipocytes [35] and also targets IL-1α in islets [36]; miR-148a-3p inhibits pro-inflammatory cytokines release via p38/MAPK signaling [37]; miR-335 reduces sepsis-dependent inflammatory response in endothelial cells via FASN [38]; overexpression of miR-125b protects against LPS-induced M1 phenotype (pro-inflammatory macrophage) to promote M2 phenotype (pro-angiogenic macrophage) [39]. Moreover, we found 5 miRNAs with pro-apoptotic properties (miR-199a modulate caspase-3 [40]; -1b target BCL-2 [41]; -143 modulate NF-κB pathway [42]; -30a target AGE-1 [43]; 148a target BCL-2 [44]) and 3 miRNAs with anti-apoptotic properties: (miR-126 promotes retinal endothelial cell survival by SetD5 regulation [45]; miR-335 modulates senescence of aging mesenchymal stromal cells [46]; miR-95-5p inhibits apoptosis and authophagy by targeting ATG7 and ATG16 [47]).

### *In silico* bioinformatic analysis of key angiogenic, inflammatory and apoptotic factors predicted to be targets of OIR-modulated miRNAs

**Fig 7:**
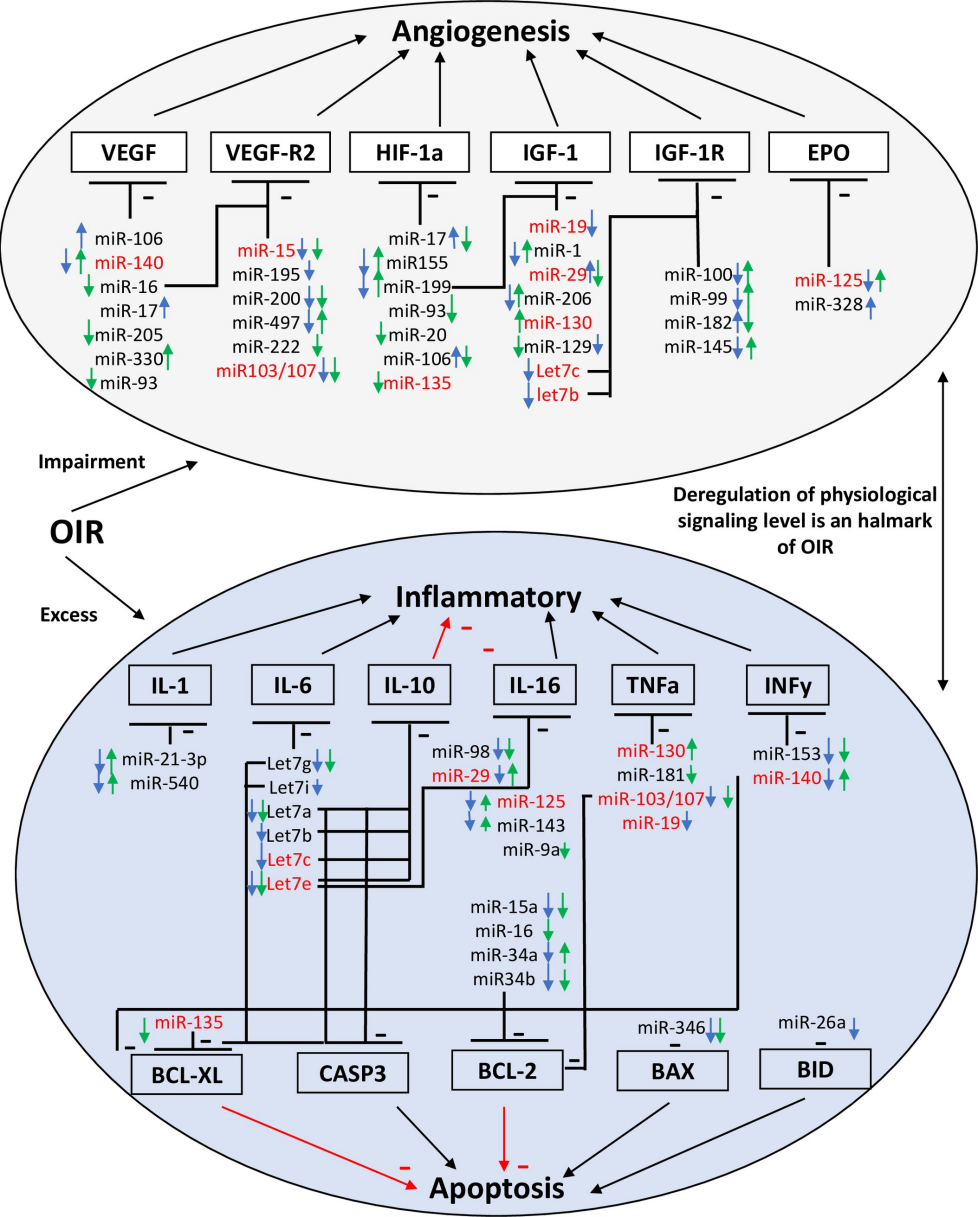
Summary of bioinformatic analysis of key angiogenic, inflammatory and apoptotic factors predicted to be targets of OIR-modulated miRNAs at P7. Connectome of predicted angiogenic, inflammatory and apoptotic targets (mRNAs) of miRNAs modulated (up or down) by >1.2 fold in OIR-subjected retina (blue arrow) and choroid (green arrow) at P7, based on bioinformatic algorithms (of miRSystem database); miRNAs identified in red refer to those that affect more than one biologic effect (i.e. angiogenesis, inflammation and/or apoptosis).

We investigated potential predictive miRNA/mRNA angiogenic, inflammatory and apoptotic targets using bioinformatics as summarized in **Fig 7**.

Six angiogenic factors (VEGF, VEGFR2, HIF-1a, IGF-1, IGF-1R, EPO), 6 inflammatory factors (IL-1, -6, -10, -16, TNFα, INFy,) and 5 apoptotic factors (BCL-2, BCL-XL, BAX, BID, CASP3) were analyzed for miRNA targeting according to algorithms of miRSystem database, which integrates seven well-known miRNA target gene predictive programs, including DIANA, miRnanda, miRBridge, PicTar, PITA, rna22 and TargetScan [18]. VEGF was the angiogenic factor with the highest number of validated miRNAs. Of these, miR-16 was highly expressed in retina and choroid from control animals but reduced in OIR at P7. miR-16 is also predicted to target VEGFR2 with a total of 6 hits on predicted target sites. On the other hand, miR-106 and miR-330 showed a great modulation profile but with lower level of expression in retinal/choroidal tissues (**S5 Table**); miRNAs predicted to regulate VEGFR2 are not validated. miR-103/107 showed a medium level of expression, but was reduced in OIR retina (retina: fc = 0.77, choroid: fc = 0.62). HIF-1a, another angiogenic target presented one validated miRNA (miR-17) that was modulated in OIR with very low level of expression in retina and choroid (**S5 Table**). miR-199, -93, -20, -135 with a medium level of expression were also predicted (≥4 hits) to target HIF-1a (**S5 Table**). Interestingly, miR-199 was very highly expressed in both retina and choroid and exhibited a significant OIR-modulated profile (retina fc = 0.02, choroid fc = 1.64). IGF-1, another important angiogenic factor with vascular protective effects in ROP, was predicted to be targeted by miR-199 and let7c, two miRs highly expressed in retina and choroid (**S5 Table**). IGF-1 type-1 receptor (IGF-1R) was predicted to be targeted by miR-182 and let7c, which are also highly expressed in retina and choroid and modulated by OIR. Only one highly expressed miR (miR-125) modulated by OIR was found to target EPO (4 hit prediction).

Among the highly expressed miRNAs in retina downregulated by >20%, and predicted to target inflammatory factor (**S6 Table**), were: miR-21-3p targeting IL-1b, the let-7 family member -g, -i, -a, -b, -c, targeting both IL-6 and the anti-inflammatory IL-10, and miR-125 and -143 targeting IL-16. In the choroid, let-7g, -a, -e target IL-6 and IL-10, let-7e and miR-9a target IL-16 and miR-181a target TNFα, were identified; interestingly, no retinal-miRNAs are found to be significantly upregulated (≥20%) for this inflammatory factor. In contrast, in the choroid, 3 highly expressed miRNAs are upregulated: miR-21-3p targeting IL-1b, miR-125 and -143, that target IL-16. The downregulated miRNA linked with predictive-apoptotic factor (**S7 Table**) are in the retina: miRNA let7i, -a, -c target simultaneously BCL-XL and CASP3, and miR-26 targets BID. In the choroid, miR-16 targets BCL-2, miR-135, and let-7g, -a, -e, -f target both BCL-XL and CASP3, while miR-30d targets only CASP3. Many other miRNAs are modulated by >20% but are not highly expressed (**Fig 7**), and some of these miRNAs have multiple targets in the same signaling pathway as well as in angiogenesis and inflammation/apoptosis, as is the case for miR-140, -15, -103/107, -135, -19, -29, -130, -125 and let7c and b (**Fig 7**).

## Discussion

Although miRNAs are recognized as critical factors in the regulation of numerous biological and cellular processes such as growth, apoptosis, inflammation, metabolism and angiogenesis [3–6], their specific participation in IRs remains to be defined. With the intention of providing a broad scope miRNA profile in different phases of OIR using a sensitive and accurate approach, we conducted the present study using NGS [14,15]. We identified a number of miRNAs in retina and choroid with altered expression during the different phases of OIR (relative to control). We found around 1000 miRNAs expressed in retinal and choroidal tissues, but we focused our attention on the most abundant miRNAs (≥1000 RPM) that were modulated by more than 20% in OIR. Our results showed around 6% (60–69 miRNA) of the total of miRNAs highly expressed in retina and choroid. This range was very similar to a previous study that evaluated the modulation pattern of miRNAs in plasma of human patients with stage 3 ROP [48], and in retina of OIR mice model [49]. RT-PCR evaluation of the 24 most modulated miRNAs validated the changes observed by NGS.

The major modulation of miRNAs in OIR occurred at P7, which corresponds to the early phase of microvascular degeneration in the retina. We identified 6 miRNAs implicated in both angiogenesis and inflammation that were downregulated in the retina by ~80% including miR-199a-5p and -3p, miR-1b, miR-126a-3p and -5p, miR-152-3p. For instance, miR-199a-3p was previously reported to be downregulated and associated with impaired microvascular function in the retina of rats with diabetic retinopathy [50] and with anti-inflammatory properties including IKK/NF-κB/IL-8 [31] and COX-2 signaling suppression [32]. miR-1 expression was reduced in a mouse model of retinal degeneration (retinitis pigmentosa) [51] and overexpression of miR-1 inhibits inflammatory response [33]. miR-152 was shown to be diminished in vitreous humour of patients with exudative AMD [52] and overexpression of miR-152 stimulates T-cell proliferation and cytokine production in gastric cancer cells lines [53]. Furthermore miR-126, implicated in vascular integrity homeostasis, embryogenesis, angiogenesis signaling, vascular repair and retinal cell survival [24,25] and inflammatory suppression [54], was reduced in peripheral blood from patients with diabetic retinopathy [55]. Intriguingly, we noticed that the miRNAs (miR-199a-5p, 126a-3p and 152-3p) associated with detrimental microvascular functions and downregulated in the retina, were upregulated (>1.6-fold) in the choroid. This infers that regulation of their expression and of their targets may differ depending upon the vascular bed; the cellular origin of these changes are also likely to differ.

Apoptosis is a critical process involved in the development of choroidal involution [19]. We identified miR-96-5p which possesses dual anti-apoptotic/pro-angiogenic functions by targeting caspase-9 in cancer cells [56] and the anti-angiogenic tyrosine-phosphatase PTPN9 [27], highly downregulated in the choroid. NGS analysis also showed that miR-9a-5p and miR-182 were the most abundant miRNAs in both retina and choroid, with high levels of expression (>100000 RPM); however, both miRNAs were exclusively altered in the choroid, but not in the retina. On the other hand, these two miRNAs have yet to be implicated in the regulation of angiogenesis, whilst miR-9a is a versatile regulator of neurogenesis [57], and is one of the most highly expressed miRNAs during brain development [58] as well as being involved in microglial inflammatory response [59]; whereas miR-182 is abundantly expressed in mammalian retina [60] as observed herein, and has been implicated to promote cell proliferation and metastasis in breast cancer [61].

Regarding the end phase of vessel degeneration at P14, miR-96-5p, miR-182, miR-183-5-5p and miR-9a were most modulated in choroid in OIR. Interestingly, it has been reported that miR-96/-183/-182 cluster is highly expressed in various types of terminally differentiating sensory neurons, including photoreceptors [62], however, its role in angiogenesis has yet to be described. On the other hand, during retinal neovascularization phase (P17), several members of the miR-30 family were altered including miR-30a-5p, miR-30e-5p, and miR-190b-5p, by ~75% downregulation and miR-30e-3p, miR-335, miR-30b-5p by ~300% upregulation. miR-30 family members are known critical regulators of development of bone, reproductive system, and adipose tissue [20], but are also involved in the pathogenesis of cardiovascular and renal disorders; while in cancer they play an important role as tumor suppressors and/or oncogenics [63]. In a study using Zebrafish as model, some member of this miRNA-30 family showed to target Delta-like 4 (DLL-4), a key regulator in vascular development and angiogenesis [64]. On the other hand, in the choroid, 50% of the miRNAs that exhibited significant modulation belong to the let-7 family. This miRNA family is the most characterized and plays a number of biological roles in ischemia [13], tumoral, and retinal angiogenesis [21]. Whereas highly expressed miR-125a-5p is involved in inhibiting endothelial cell proliferation by targeting RTEF-1 genes [65] and in the constitutively activation of NF-κB pathway [66], which may suggest a role of this miRNA in choroidal involution.

The expression pattern of the 9 most modulated miRNAs identified was complex and characterized by bi- and tri-phasic modulation curves that varies with time and ocular origin (retina or choroid). We documented the targets for each of the miRNAs exhibiting major changes. Our research in the literature showed that miR-1b-5p, -30a-5p, -126a-5p, 96-5p have pro-angiogenic functions [22,23,25,27]. For instance, miR-1b-5p is known to control postnatal cardiomyocyte proliferation by targeting Spred1 [22], and miR-30a-5p regulates endothelial tips cell formation and arteriolar branching by modulating Notch signaling pathway [23]. On the other hand, miR-126a-5p, an endothelial specific miRNA, is well-known to be required for vascular integrity, embryogenesis and angiogenic activation signaling [24,25]. While miR-96-5p, a miRNA induced in hypoxic conditions, exerts pro-angiogenic functions in tumorigenesis [26,27]. Other miRNAs such as miR-199a-5p and -125b-5p act as negative regulators of angiogenesis [29,30]. For instance, miR-199a-5p is known to inhibits VEGF-induced tumorigenesis [67] and suppresses human bladder cancer cell metastasis by targeting C-C chemokine receptor type 7 (CCR7) [68]; this miRNA is also documented to target ETS-1 and suppresses HMEC angiogenesis by inhibiting VEGF and HGF signaling [29]. miR-125b-5p expression inversely correlates with VEGF expression [30] and miR-125a are involved in the regulation of hematopoietic stem cell number [69]. In the case of miR-143-3p, -148a-3p, -335 they were reported with opposing functions/effects on angiogenesis [70–75]. Finally, we performed bioinformatic analysis of putative angiogenic key factors (VEGF, VEGFR2, HIF-1a, IGF-1, IGF-1R, EPO) predicted to be targeted by altered miRNAs during OIR. miR-199 (fc = 0.02), miR-182 (fc = 1.25) and miR-125 (fc = 0.43) predicted to target at least one of the following angiogenic factors, IGF-1, IGF-1R and EPO. miR-16 and miR-let-7c targeted two factors at once, VEGF and VEGFR2 for the former, and IGF-1 and IGF-1R for the latter. Moreover, some OIR-modulated miRNAs are predicted to target simultaneously angiogenesis and inflammatory/apoptotic processes as is the case for Let-7c and -e, miR-135, -29, 130, -103/107, -19 and -140.

The present study focused on retinal and choroidal miRNA upon exposure to hyperoxia. Nonetheless, the expression profile of miRNAs in rat has been reported to be modulated by hyperoxia-induced oxidant stress in other tissues [76] as well as in plasma [77]. An analysis of more than 100 scientific articles reveals that the expression pattern of miRNAs is modified under such oxidative stress in different cell types and tissues, and suggests specific miRNAs as circulating biomarkers [78].

To our knowledge, this is the first study to describe the alterations on miRNA expression profiling in both retinal and choroidal tissues during genesis of OIR by using NGS technology. This is also the first comparative study exploring the effects of hyperoxia on miRNA modulation and its correlation with vessel degeneration in OIR, complementing a previous a previous report on miRNAs-profiling in a mouse model of proliferative retinopathy, where retinal (but not choroidal) miRNAs were identified, using an array rather than NGS, and performed only during the NV phase [79]. Although there are differences between species regarding vulnerability to OIR [80,81], the present study utilizes an established reproducible model of OIR in Sprague-Dawley rats [82], which complements other works [79]. In recent years, clinical studies in humans and animal studies have identified a large-spectrum of deregulated miRNAs involved in development and progression of several diseases such cancer, cardiovascular, metabolic and degenerative pathology [3–5,7,8]. In this regard miRNA-based therapy may have potential as shown with angiomiRs for tumor angiogenesis and cardiovascular revascularization [4,11]. Overexpression or inhibition of specific miRNAs using miRs mimic or antagomir therapy, allows to determine functionally relevant miRNAs during ontogeny and pathology, accordingly potentially slowing down and/or reversing the development of targeted disorders [3,7,12,13]. The present study provides important cues for IRs and specifically OIR, by establishing a solid framework to further explore the characterization of specific miRNAs involved in microvascular modulation process during retinopathy, that can lead to the development of potential miRNA-based therapy, as suggested based on preclinical studies [83–85].

## Supporting information

S1 Fig Effect of hyperoxia on retinal and choroidal vasculature. Representative images of flat-mounted retinas stained with lectin revealing (A) vasculature of normoxic (CTL) and OIR-subjected rats at P14 and P17, and (B) choroid thickness.
(TIF)

S1 Table Summary of next generation sequencing raw reads generated. (TIF)

S2 Table Analytic summary of 9 most significantly altered miRNAs in OIR associated with angiogenesis (Table 1 of 2). (TIF)

S3 Table Analytic summary of 9 most significantly altered miRNAs in OIR associated with angiogenesis (Table 2 of 2). (TIF)

S4 Table Interaction of the 9 most significantly modulated miRNAs in OIR with inflammatory and apoptotic processes. (TIF)

S5 Table Bioinformatic analyses of angiogenic factors predicted to be targeted by OIR-modulated miRNAs during vascular degeneration (at P7). (TIF)

S6 Table Bioinformatic analyses of inflammatory factors predicted to be targeted by OIR-modulated miRNAs during vascular degeneration (at P7). (TIF)

S7 Table Bioinformatic analyses of apoptotic factors predicted to be targeted by OIR-modulated miRNAs during vascular degeneration (at P7). (TIF)

S8 Table List of most upregulated and downregulated miRNAs in OIR condition at P7, P14 and P17 in the retina and choroid. OIR and CTL Values are RPM and fold change (FC).
(TIF)
