# Supplementary figures and images for: MicroRNA expression profile in retina and choroid in oxygen-induced retinopathy model
Source: PLoS One. 2019 Jun 12;14(6):e0218282. doi: 10.1371/journal.pone.0218282 (PMC6561584; doi:10.1371/journal.pone.0218282)

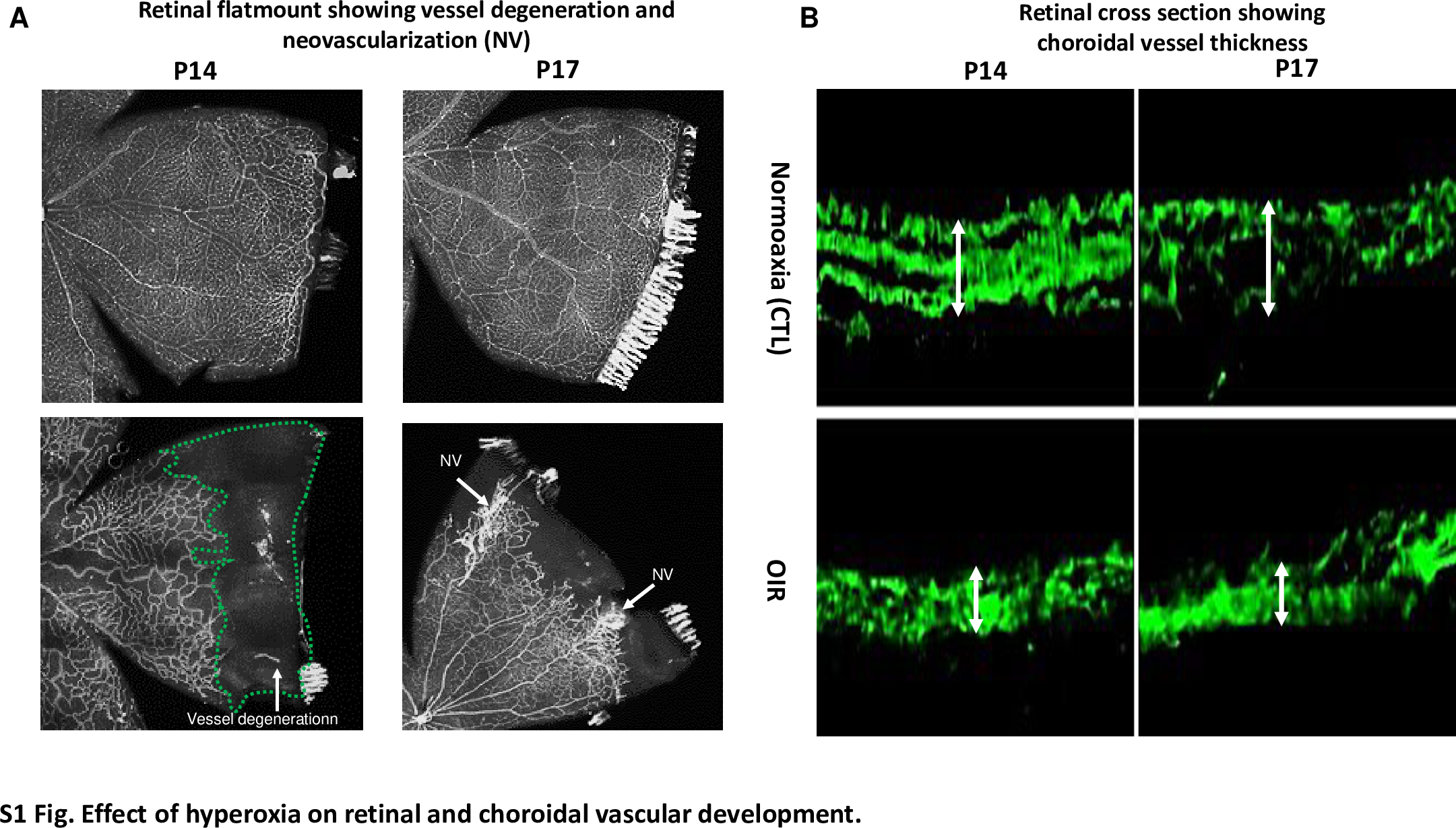

Supplement: S1 Fig — Representative images of flat-mounted retinas stained with lectin revealing (A) vasculature of normoxic (CTL) and OIR-subjected rats at P14 and P17, and (B) choroid thickness. (TIF) [file pone.0218282.s001.tif]

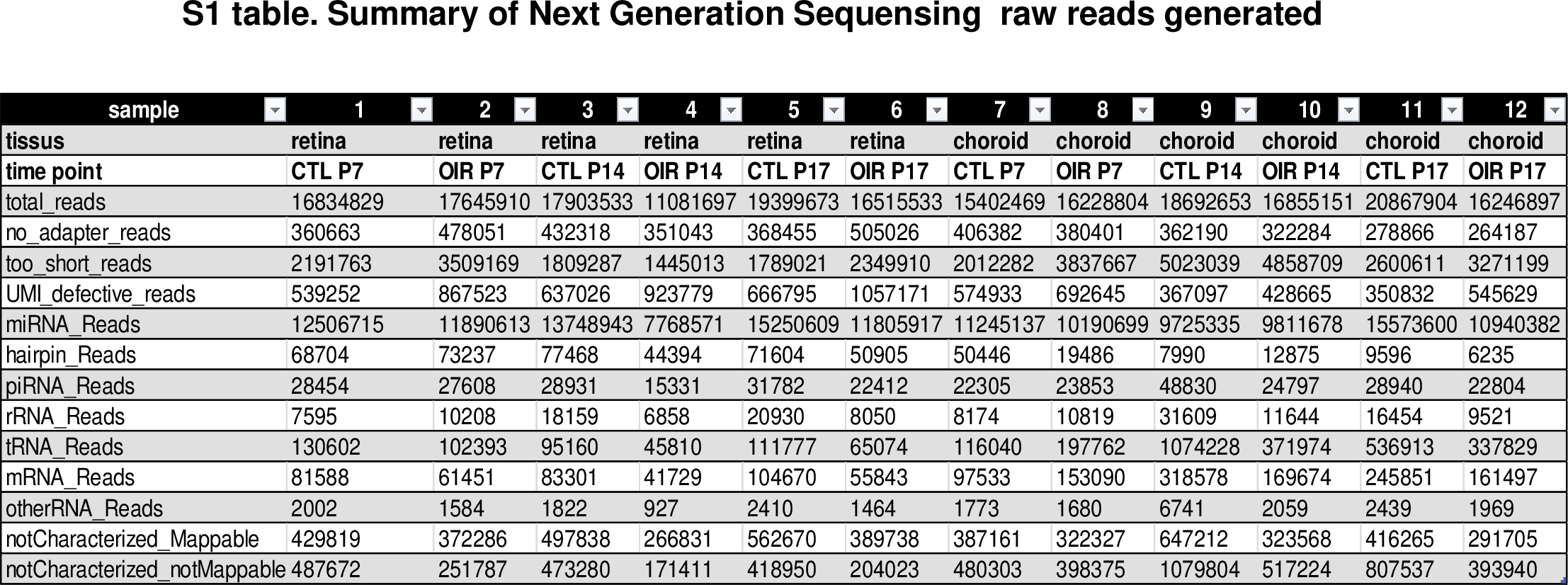

Supplement: S1 Table — (TIF) [file pone.0218282.s002.tif]

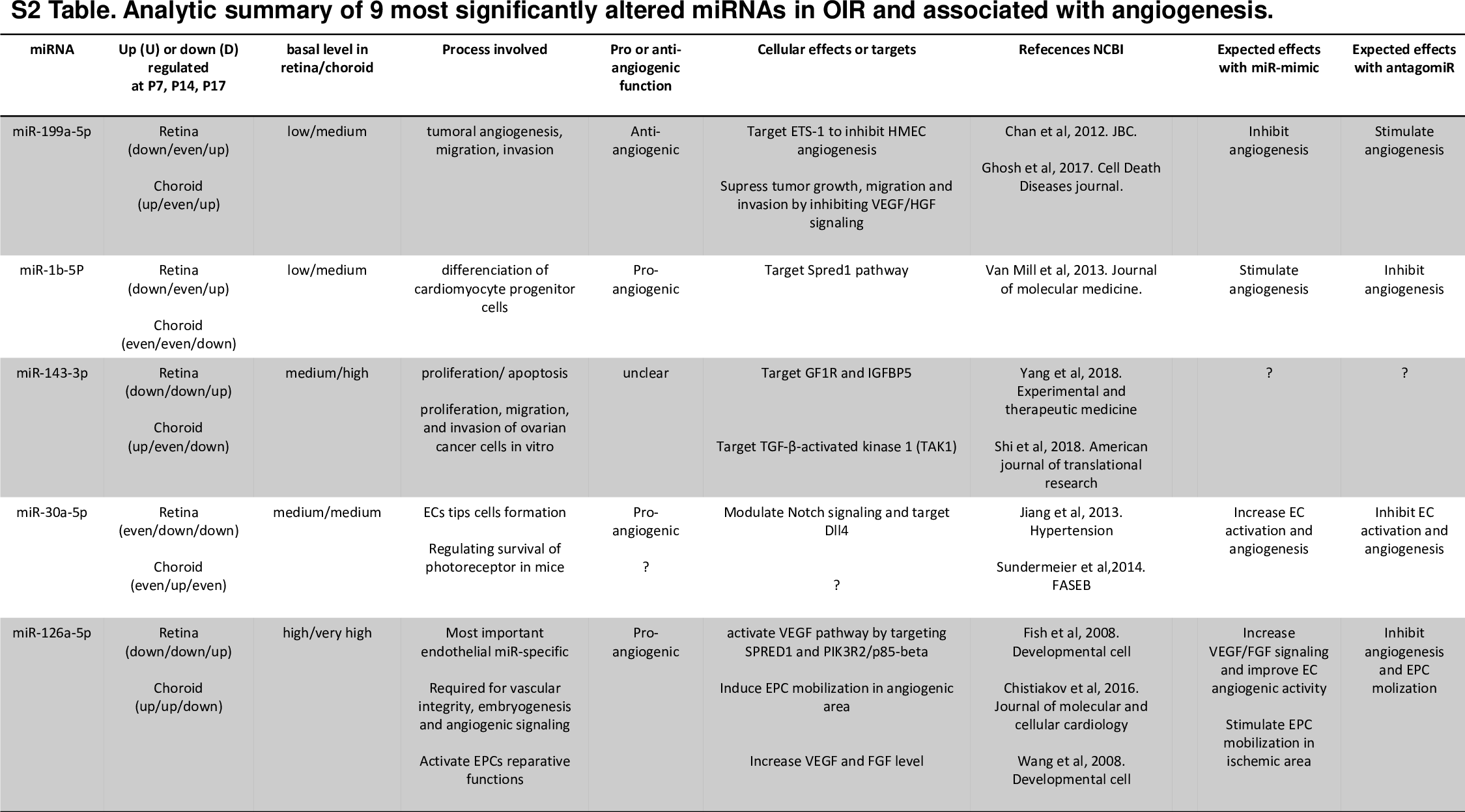

Supplement: S2 Table — (TIF) [file pone.0218282.s003.tif]

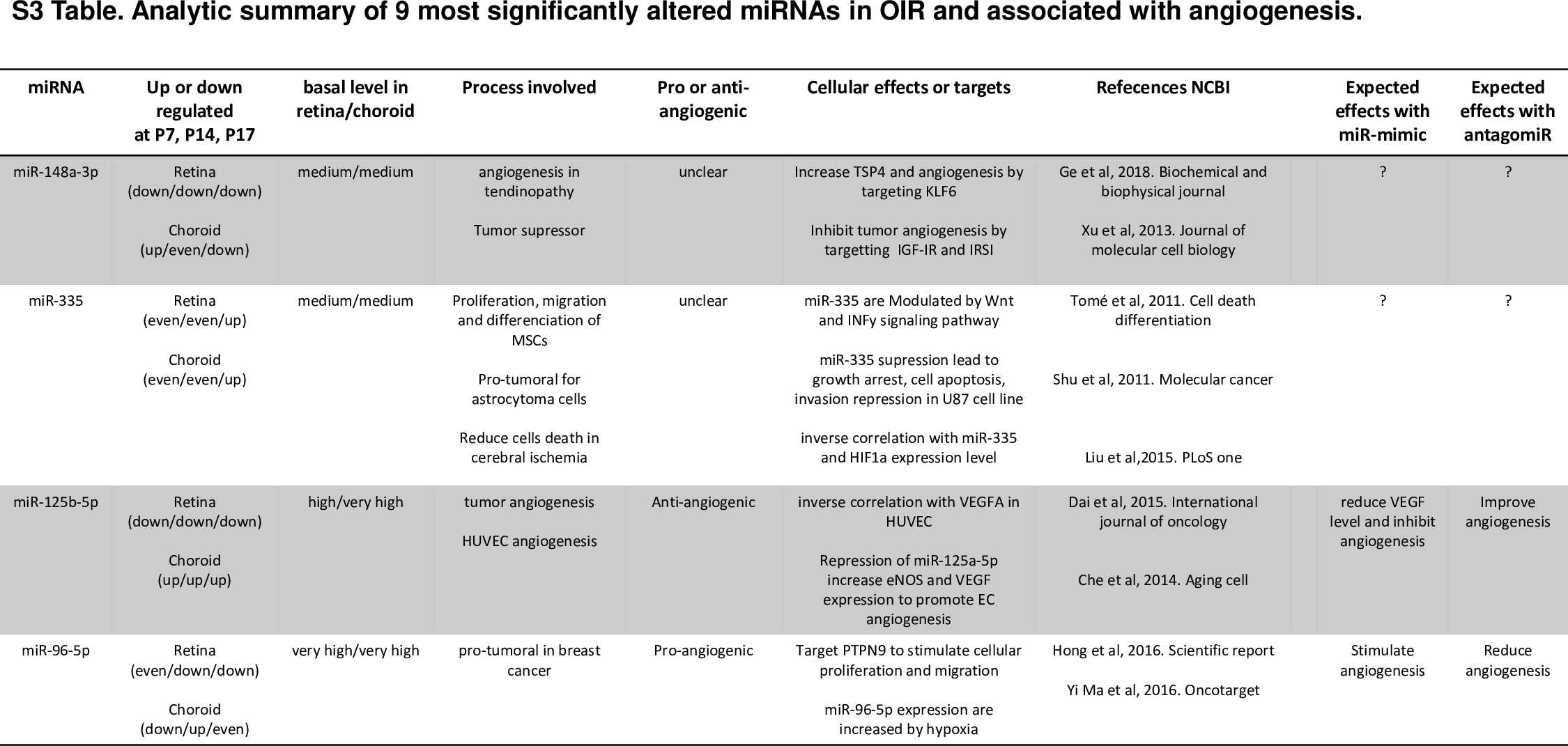

Supplement: S3 Table — (TIF) [file pone.0218282.s004.tif]

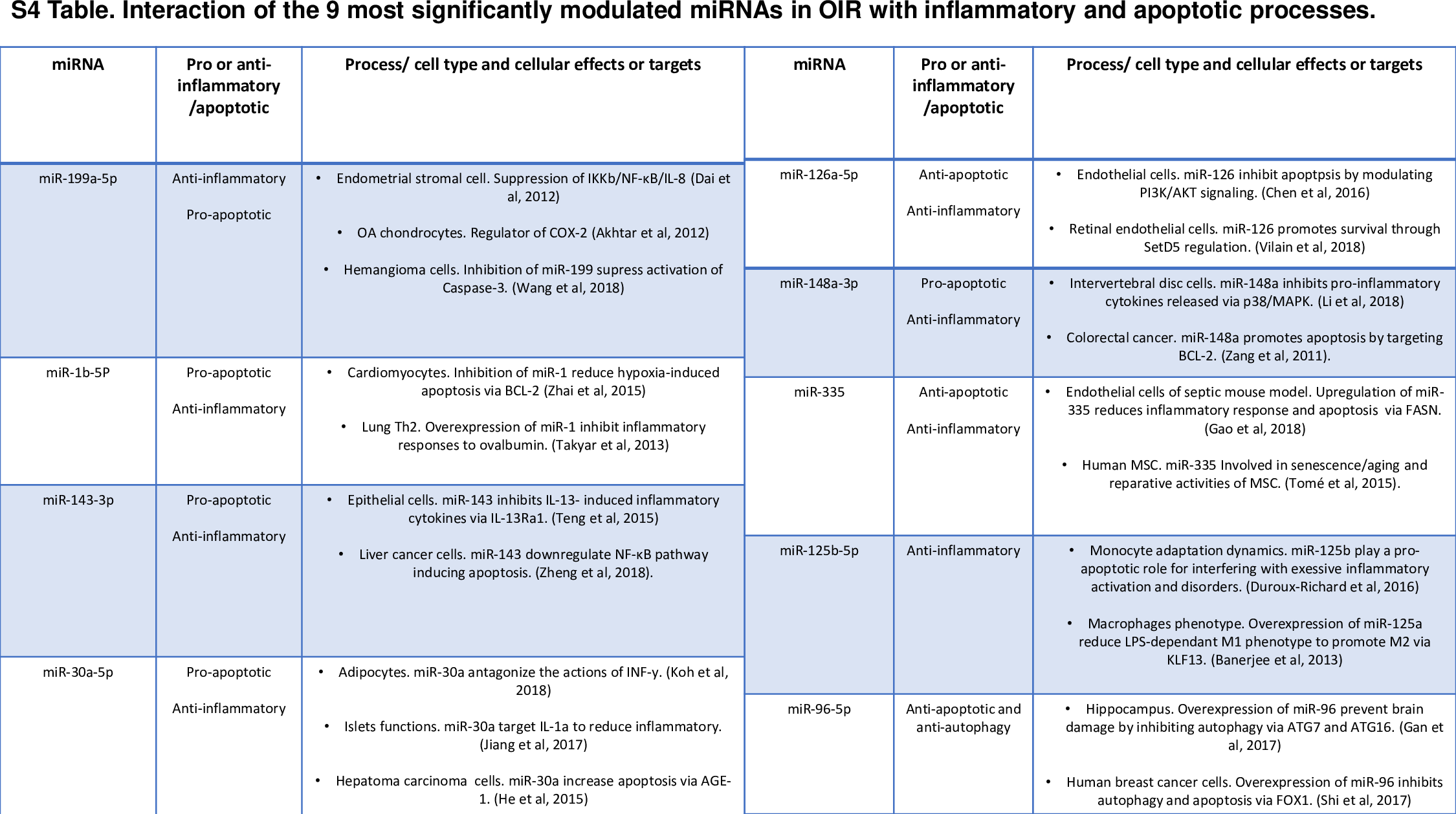

Supplement: S4 Table — (TIF) [file pone.0218282.s005.tif]

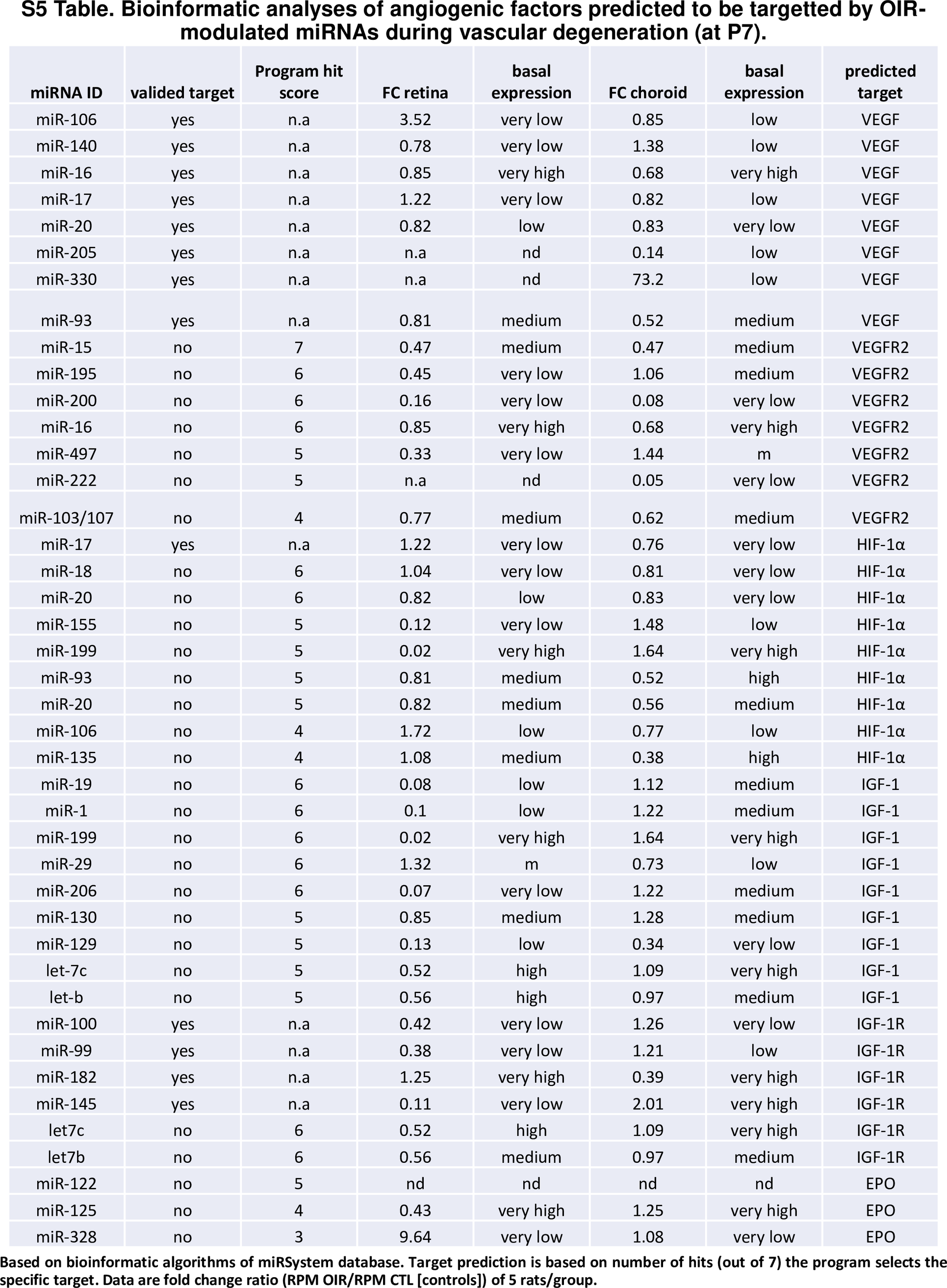

Supplement: S5 Table — (TIF) [file pone.0218282.s006.tif]

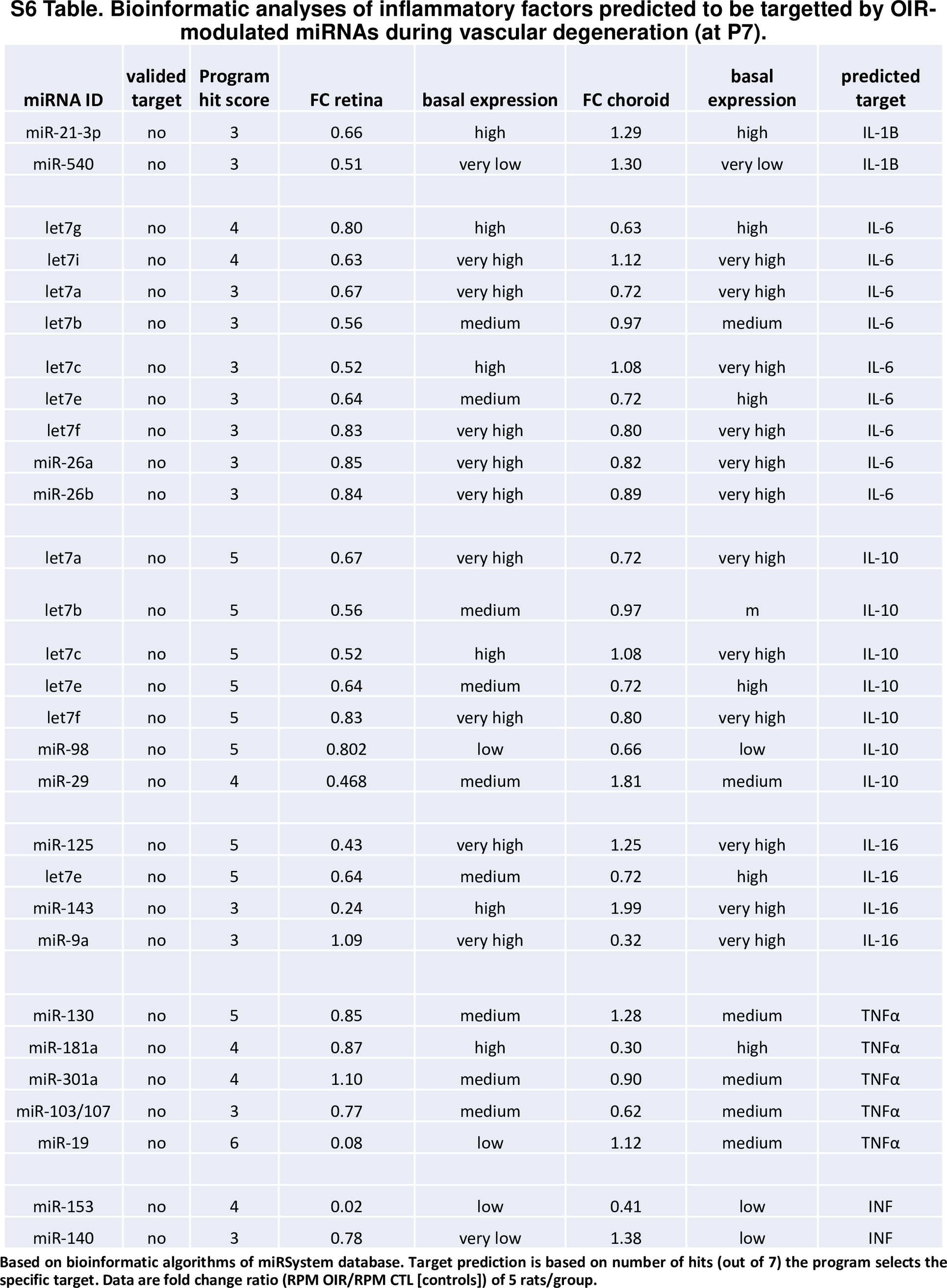

Supplement: S6 Table — (TIF) [file pone.0218282.s007.tif]

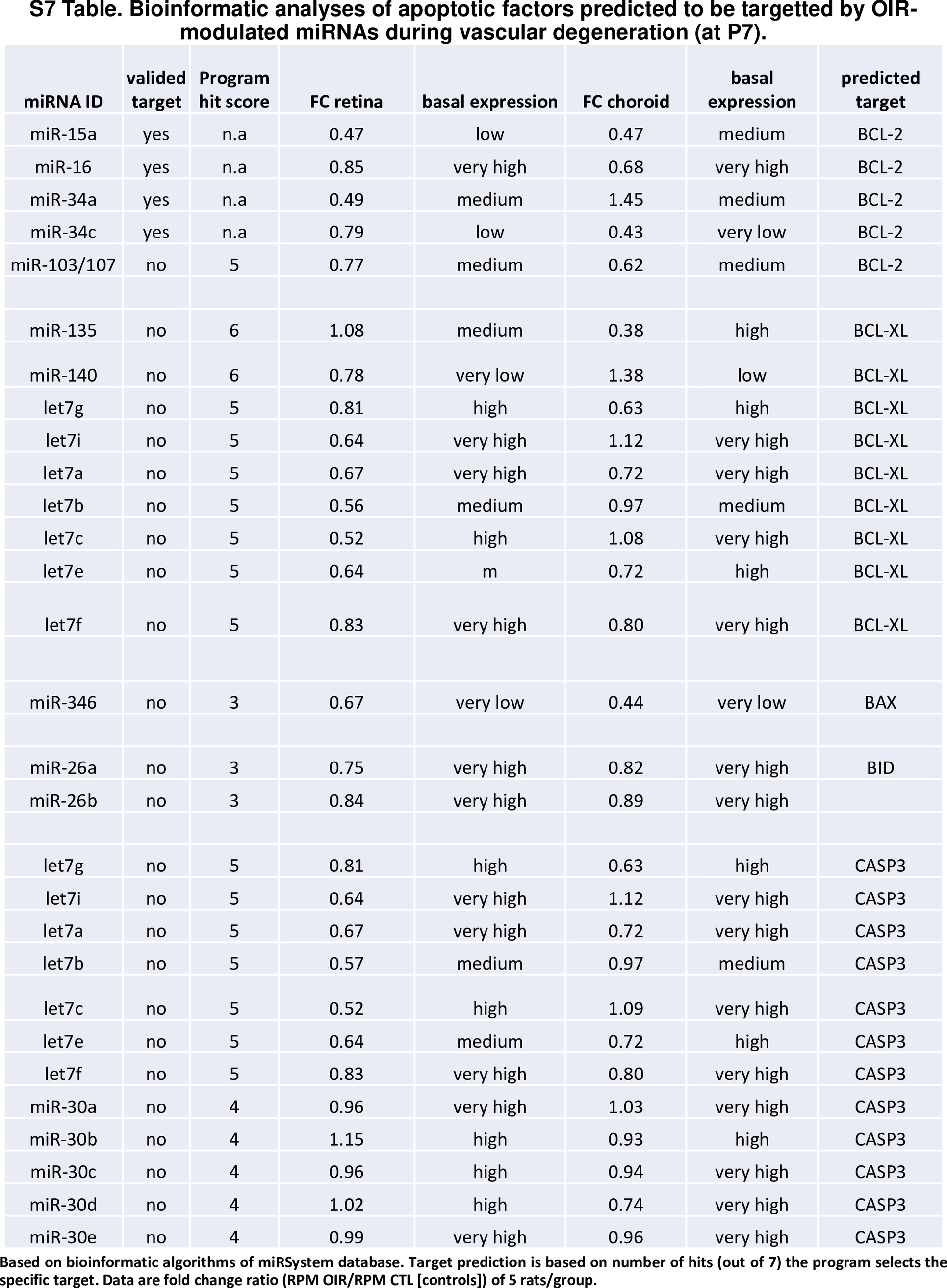

Supplement: S7 Table — (TIF) [file pone.0218282.s008.tif]

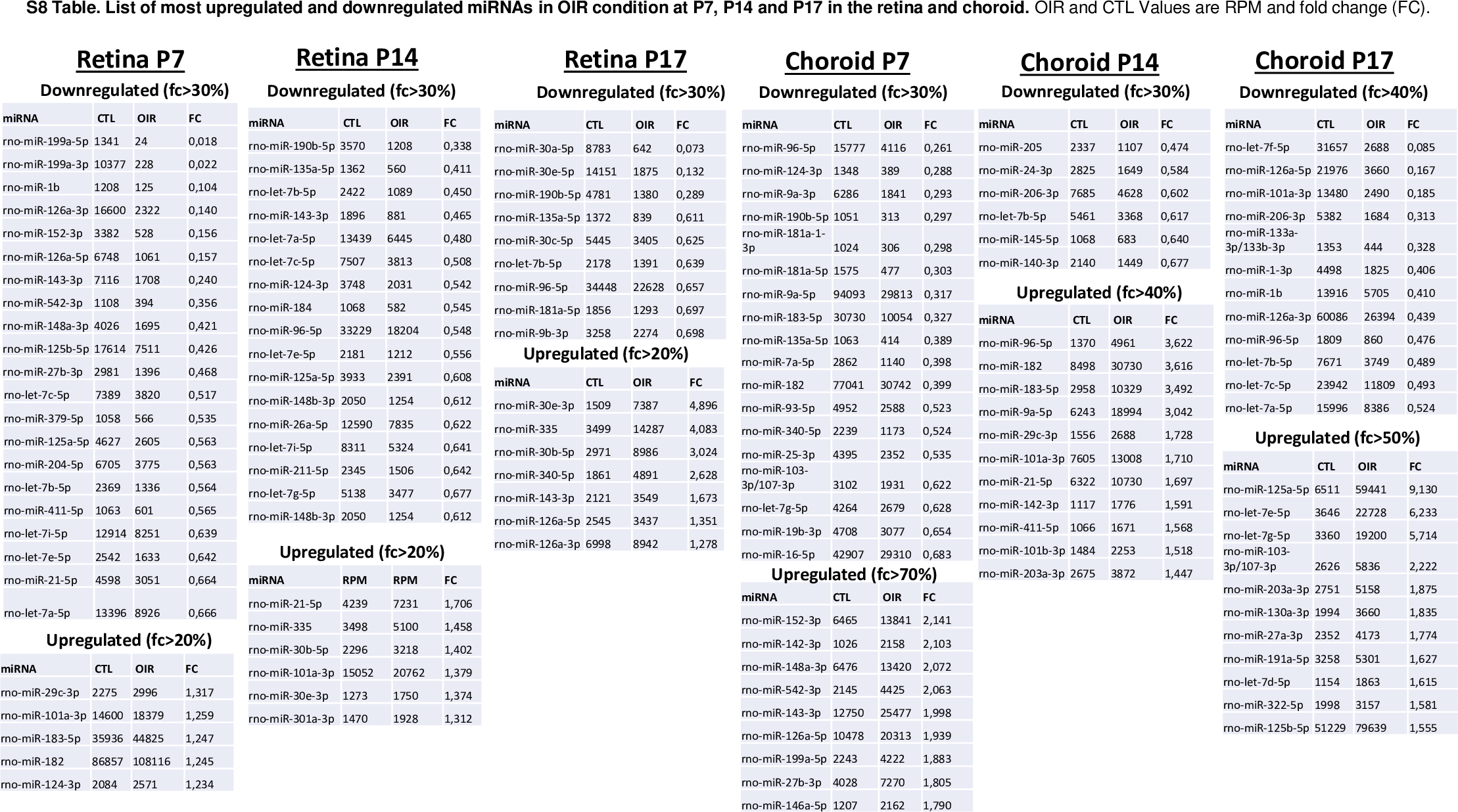

Supplement: S8 Table — OIR and CTL Values are RPM and fold change (FC). (TIF) [file pone.0218282.s009.tif]
